# Additional moults into ‘elongatus’ males in laboratory-reared *Polydesmus angustus* Latzel, 1884 (Diplopoda, Polydesmida, Polydesmidae) – implications for taxonomy

**DOI:** 10.3897/zookeys.156.2045

**Published:** 2011-12-20

**Authors:** Jean-Francois David, Jean-Jacques Geoffroy

**Affiliations:** 1 Centre d’Ecologie Fonctionnelle & Evolutive, CNRS, 1919 Route de Mende, F-34170 Montpellier cedex 5, France; 2 Equipe Evoltrait, Dept. Ecologie & Gestion de la Biodiversité, Museum National d’Histoire Naturelle, 4 Avenue du Petit-Chateau, F-91800 Brunoy, France

**Keywords:** post-embryonic development, anamorphosis, sexual maturity, stadium number variation, taxonomy

## Abstract

The number of stadia during post-embryonic development is supposed to be fixed in most species of the millipede order Polydesmida. For the first time since 1928, additional moults were observed in two males of *Polydesmus angustus* Latzel, 1884 reared in the laboratory. These ‘elongatus’ males sensu Verhoeff reached stadium IX instead of stadium VIII, with addition of a further podous ring (32 pairs of legs). One male had well-developed gonopods at stadium VIII, which regressed at stadium IX; the other had no gonopods at stadium VIII, which developed at stadium IX. The two cases correspond to the ‘regressionis’ and ‘progressionis’ forms described by Verhoeff in *Polydesmus complanatus* (Linnaeus, 1761), which confirms entirely his results. Additional moults appear to be associated with small body sizes and possible underlying mechanisms are discussed. Comparisons between millipede orders indicate that post-embryonic development is less strictly canalized in Polydesmida than in Chordeumatida. This implies that the adult number of body rings is of limited taxonomic value in Polydesmida and should not be viewed as a character of generic importance.

## Introduction

In millipedes (Diplopoda), post-embryonic development occurs basically by anamorphosis, i.e. the number of body rings is small at birth (e.g., four podous rings bearing three pairs of legs, two apodous rings, plus the telson) and increases progressively at each moult. However, the relationships between developmental stages (= stadia) and sexual maturity vary greatly depending on the order (Enghoff et al. 1993). In most millipede orders, adults occur in several stadia within a species, either because the stadium at which maturity is reached varies among individuals, or because adults undergo further moults. In addition, adults in a given stadium often have a variable number of body rings (e.g. in Julida, Polyzoniida, Spirobolida, Spirostreptida). By contrast, development is much more canalized in the order Polydesmida, in which there is generally only one adult stadium in each species, with a fixed number of body rings (Mesibov 2011). In most polydesmidan species, adults are in stadium VIII, with the head, 18 podous rings, 1 apodous ring and the telson (in abbreviated form: 18+1+T); in other species, adults are in stadium VII (17+1+T); in a few species, the stadium and ring number of adults differ between the sexes, but with no intra-sex variation. In spite of obvious exceptions, e.g. in the genus *Devillea* Brölemann, 1902 (Xystodesmidae), in which adults probably occur in several stadia (Enghoff et al. 1993), the number of body rings in the adult is considered as fixed in most Polydesmida and is sometimes used in taxonomy (e.g. Djursvoll et al. 2000).

Surprisingly, Verhoeff (1916, 1928) reported the occurrence of an additional moult in a number of male and female *Polydesmus complanatus* (Linnaeus, 1761) (Polydesmidae) reared in the laboratory. Whereas adults of this species are normally in stadium VIII (18+1+T), some individuals were observed in stadium IX with a further podous ring (19+1+T). Verhoeff (1928) was convinced that the existence of these ‘elongatus’ specimens shed light on the evolution of ring numbers in millipedes and used it as an argument in his controversy with Brolemann (1921) over elongation vs. contraction. However, this additional moult in a polydesmid has sometimes been regarded sceptically, and Enghoff et al. (1993: p. 153) did not exclude the possibility of Verhoeff’s mistakes in the counting of rings. More recently, intraspecific variability in the ring number of adult males was demonstrated in another polydesmidan millipede, the pyrgodesmid *Muyudesmus obliteratus* Kraus, 1960 (Adis et al. 2000).

Herein, the reality of the phenomenon observed by Verhoeff in *Polydesmus* is confirmed for the first time since 1928. During laboratory studies on another, closely related species, *Polydesmus angustus* Latzel, 1884, two cases of moult into stadium IX were observed in males. In the present paper, we first describe the conditions under which these moults occurred and some characteristics of the males before and after moulting, we briefly discuss possible underlying mechanisms, and then highlight implications of intraspecific variability in ring number for the taxonomy of Polydesmida.

## Material and methods

### Study species

The flat-backed millipede *Polydesmus angustus* is widespread in north-western Europe, west of the range of *Polydesmus complanatus* (Kime and Enghoff 2011). Its post-embryonic development consists of eight stadia, which can be determined by counting the number of body rings, and the sexes can be distinguished from stadium IV onwards (Enghoff et al. 1993). Towards the end of each immature stadium, millipedes build a chamber made of earthy faecal material, in which they coil during moulting. The last stadium is the adult (18+1+T), which dies after the breeding season. The life cycle is completed in either one or two years depending on the individuals. Under mean seasonal conditions, development time from egg hatch to adult emergence lasts about 10 months for annual individuals, plus a further 3 months in aestivation for biennial individuals (David et al. 1999).

### Laboratory rearing

During experimental studies on the biology and ecology of *Polydesmus angustus*, hundreds of specimens were reared throughout their life cycle in the laboratory. Broods that were produced by adults from a field population living at Brunoy, 20 km south-east of Paris, were kept in lidded, transparent plastic boxes containing 1 cm of sieved soil and moist leaf litter. The boxes were placed in incubators fitted with a glass door and exposed to natural daylight. Temperature followed the mean monthly temperatures of the region of origin, with a daily thermoperiod of 4°C (David et al. 1999). The young from each brood were reared in groups up to stadia IV–V, sexed and then kept at a low population density to be monitored individually. Some individuals were fed on leaf litter alone throughout development, while others received a monthly pinch of dry yeast in addition to leaf litter, which greatly improves growth and female fecundity (David and Celerier 1997). Under those laboratory conditions, additional moults were observed in two males, one of which was reared with yeast and the other without.

## Results

**Figure 1.**
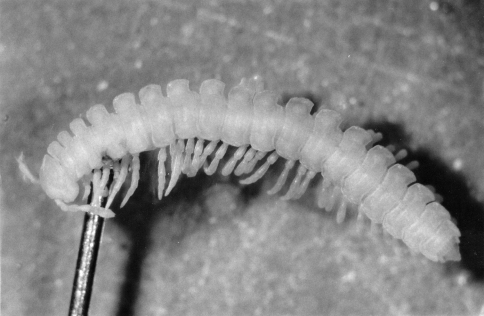
Dorsal view of a *Polydesmus angustus* male of stadium IX after its death at the age of 19 months. The trunk consists of 19 podous rings – bearing 32 pairs of legs plus regressed gonopods (8th leg-pair) – and one apodous ring anterior to the telson.

The first ‘elongatus’male hatched in late August from eggs produced by field-collected parents (first generation in the laboratory). It was fed on leaf litter without dry yeast and emerged as a small adult (stadium VIII) in mid-September, at the age of 12 months. Its live weight was 15.3 mg, which is usually the weight of a stadium VII specimen in the field. The 8th leg-pair was transformed into apparently normal, well-developed gonopods. The male was left unmated and received a pinch of yeast in early October. It coiled into a moulting chamber on October 27th and then emerged as stadium IX (19+1+T) on November 18th. Its live weight was 22.4 mg. Although the examination of the exuvia confirmed that its gonopods were fully formed in stadium VIII, with all the characteristics of *Polydesmus angustus*, they were transformed regressively at stadium IX. The coxites were strongly developed, the coxal hooks clearly visible, but the telopodites were even more simplified and atrophied than in the figures shown by Verhoeff (1928: p. 691). This type of additional moult with regression of gonopods is similar to the ‘regressionis’ form described by Verhoeff (1916). This ‘elongatus’ male overwintered a second time and died in late March (Fig. 1).

The second male hatched in June from eggs produced by laboratory-reared parents (second generation in the laboratory). It was fed on leaf litter supplemented with yeast and emerged as stadium VIII on May 3rd, at the age of 10 months. It had the normal number of rings (18+1+T) but was extremely small. Its live weight was 9.2 mg, which is usually the weight of a stadium VI specimen in the field. It had no gonopods, the 8th leg-pair being replaced by small buds, as is usually the case in immature males of stadium VII. This male coiled into a moulting chamber in mid-May. Although it was accidentally frozen before emergence due to an incubator failure, the moulting process had already reached an advanced stage. An examination of the specimen dead before emergence clearly showed that it was about to give a male in stadium IX (19+1+T) with 32 leg-pairs and developed gonopods. This type of additional moult with appearance of gonopods is similar to the ‘progressionis’ form described by Verhoeff (1916).

## Discussion

It is clear that the number of stadia is not completely fixed in millipede species of the family Polydesmidae. Additional moults described by Verhoeff (1916, 1928) in *Polydesmus complanatus* have been confirmed in adult males of *Polydesmus angustus*. In the latter species, it is a very rare phenomenon, which was observed in only two males and no females. Nevertheless, two modalities corresponding to Verhoeff’s ‘regressionis’ and ‘progressionis’ forms have been confirmed, which lends considerable credibility to all the results reported by Verhoeff (1928). This also suggests that the additional moults briefly mentioned by Stephenson (1961) in a number of males of *Brachydesmus superus* Latzel, 1884 from the field, may not necessarily be due to confusion of species.

### Possible underlying mechanisms

In this study, the two ‘elongatus’ males with 19 podous rings were obtained under controlled laboratory conditions and did not experience environmental stress in terms of temperature, humidity and photoperiod. However, an obvious difference between these males and normally developing animals was their small body size at stadium VIII. Similarly, the first ‘elongatus’ specimens obtained by Verhoeff (1916) were from a poorly-fed brood that yielded very small adults (the so-called ‘forma nana’), and most of those he obtained later were less than 20 mm long at stadium VIII (Verhoeff 1928). Body size may thus be critical for the appearance of ‘elongatus’ specimens. A small size can result from poor quality food (David and Celerier 1997), although this is not a valid explanation for the smaller ‘elongatus’ male obtained in *Polydesmus angustus*, which was reared on leaf litter supplemented with yeast. In an experiment with a species of the order Spirobolida in which the mode of anamorphosis is very different from that of Polydesmida, Berns and Keeton (1968) showed that semi-starvation resulted in smaller juveniles, and these underwent a greater number of moults before maturity than well-fed individuals.

Hormone imbalance is undoubtedly involved in the occurrence of additional moults, and body size may play a role in this respect. Although little is known about the hormonal control of development in millipedes (Descamps et al. 1990; Hopkin and Read 1992), hypotheses based on knowledge from other arthropod classes can be put forward. In insect species with variable numbers of instars, such as the Lepidoptera *Manduca sexta* (Linnaeus, 1763) (Sphingidae) and *Malacosoma disstria* Hübner, 1820 (Lasiocampidae) (Kingsolver 2007; Etile and Despland 2008), further larval moults occur only in small individuals, because attainment of a critical weight is the signal that stops juvenile hormone production (Davidowitz et al. 2003). A similar mechanism can be hypothesized for the additional moult in the ‘progressionis’ form of *Polydesmus*, which is typically the occurrence of a further immature stadium during development.

The ‘regressionis’ form in males is more difficult to interpret. In this case, small males reach stadium VIII with the normal number of rings and well-developed gonopods. However, according to Verhoeff (1928), these males are unable to breed because other adult characters are missing, especially the male openings on the coxae of the second pair of legs. These characters appear in stadium IX, while the gonopods are transformed regressively. A mixture of development and regression of secondary sexual characters during the additional moult is difficult to interpret in terms of hormonal control.

It is clear, however, that the additional moult with regression of gonopods in *Polydesmus* is not akin to periodomorphosis (Verhoeff 1923). This phenomenon, which is well known in the order Julida, involves the appearance of intercalary males with regressed sexual characters between two adult stadia (Enghoff et al. 1993). If *Polydesmus* males in stadium VIII are not sexually mature despite their well-developed gonopods, their moult to stadium IX is by no means the first stage in periodomorphosis (Verhoeff 1928; Sahli 1968). While some secondary sexual characters regress, others develop in stadium IX, so that ‘regressionis’ males are quite different from true intercalary males. Moreover, the next stage in periodomorphis, i.e. a further moult into mature males of stadium X, has never been observed in *Polydesmus*.

## Taxonomic implications

The confirmation that additional moults can occur in Polydesmida, the post-embryonic development of which is generally assumed to consist of a fixed number of stadia, has implications for taxonomy. In *Polydesmus*, adults with a further body ring are quite capable of surviving and Verhoeff (1928) even showed that ‘elongatus’ females of *Polydesmus complanatus* were able to breed. Although such individuals have not been reported so far in field populations of *Polydesmus*, they might be encountered besides normally developing adults under specific ecological conditions, especially in populations composed of small individuals. Clearly, it is biologically possible. Therefore polydesmidan millipedes that have very similar characteristics, particularly the male gonopods, but different numbers of rings in the adult, might belong to the same species. They should not be automatically classified as distinct genera. For example, Demange (1970) stressed that the polydesmids *Brachydesmus proximus* Latzel, 1889 (17+1+T) and *Polydesmus geochromus* Attems, 1952 (18+1+T) have virtually identical gonopods; the paradoxosomatids *Paradoxosoma granulatum* Daday, 1889 (17+1+T) and *Trachydesmus simonii* Daday, 1889 (18+1+T) also have virtually identical gonopods. Distinctions at the generic level for such closely related organisms were criticized (Jeekel 1968; Demange 1970), and the latter author correctly argued that these differences in ring number could reflect environmentally induced variation within a single species. In the recent scientific literature, Shelley (2000) chose the option to classify adult males with 17 and 18 podous rings in the same sphaeriodesmid species, *Desmonus pudicus* (Bollman, 1888). At most, such differences could reflect speciation, assuming that each ring number has already been fixed in reproductively isolated populations. But the number of rings as such should no longer be viewed as a character of generic importance in Polydesmida. Its use in phylogenetic analyses, working on the assumption that this character shows no intraspecific variation (Djursvoll et al. 2000), perpetuates divisions which may be artificial, such as the genera *Brachydesmus* (17+1+T) and *Polydesmus* (18+1+T).

## Conclusion

Enghoff et al. (1993) coined the term teloanamorphosis to describe a mode of anamorphosis in which the number of moults and the number of rings added at each moult are fixed within each species (or each sex of a given species). In millipedes, this type of post-embryonic development is characteristic of the orders Chordeumatida and Polydesmida, with some interspecific variation in the number of stadia to maturity. However, there appears to be a difference between the two orders. Whereas no additional moults have ever been mentioned in the Chordeumatida, the data available to date show that there are various degrees of intraspecific variability for the stadium number in Polydesmida, at least in *Devillea*, *Muyudesmus* and *Polydesmus*, and possibly in other genera mentioned above. The number of body rings in the adult appears therefore to be of more limited taxonomic value in this order.

## References

[B1] Adis J Golovatch SI Wilck L Hansen B (2000) On the identities of *Muyudesmus obliteratus* Kraus, 1960 versus *Poratia digitata* (Porat, 1889), with first biological observations on parthenogenetic and bisexual populations (Diplopoda: Polydesmida: Pyrgodesmidae). In: Wytwer J Golovatch SI (Eds). Progress in Studies on Myriapoda and Onychophora. Fragmenta Faunistica (Suppl.) 43: 149–170.

[B2] Berns MW Keeton WT (1968) Effects of semi-starvation on growth and morphogenesis during the larval stages of a common milliped, *Narceus annularis* (Raf.). Biological Bulletin 135: 454-465. doi: 10.2307/1539708

[B3] Brolemann HW (1921) Principe de contraction contre principe d’élongation. Bulletin de la Société d’Histoire Naturelle de Toulouse 49: 340-357.

[B4] David JF Celerier ML (1997) Effects of yeast on the growth and reproduction of the saprophagous millipede *Polydesmus angustus* (Diplopoda, Polydesmidae). Biology and Fertility of Soils 24: 66-69. doi: 10.1007/BF01420222

[B5] David JF Celerier ML Geoffroy JJ (1999) Periods of dormancy and cohort-splitting in the millipede *Polydesmus angustus* (Diplopoda: Polydesmidae). European Journal of Entomology 96: 111-116.

[B6] Davidowitz G D’Amico LJ Nijhout HF (2003) Critical weight in the development of insect body size. Evolution & Development 5: 188-197. doi: 10.1046/j.1525-142X.2003.03026.x 12622736

[B7] Demange JM (1970) Myriapodes diplopodes de Madère et des Açores. Boletim do Museu Municipal do Funchal 25: 5-43.

[B8] Descamps M Sahli F Jamault-Navarro C Caplet J (1990) Morphology, histology, and ultrastructure of cephalic neurohemal organs and their roles in morphogenetic processes in Myriapoda. In: Gupta AP (Ed). Morphogenetic Hormones of Arthropods, Vol. 1, Part 2. Rutgers University Press, New Brunswick: 195-232.

[B9] Djursvoll P Golovatch SI Johanson KA Meidell B (2000) Phylogenetic relationships within *Polydesmus* sensu lato (Diplopoda: Polydesmidae). In: Wytwer J Golovatch SI (Eds). Progress in Studies on Myriapoda and Onychophora. Fragmenta Faunistica (Suppl. ) 43: 37–59.

[B10] Enghoff H Dohle W Blower JG (1993) Anamorphosis in millipedes (Diplopoda) – the present state of knowledge with some developmental and phylogenetic considerations. Zoological Journal of the Linnean Society 109: 103-234. doi: 10.1111/j.1096-3642.1993.tb00305.x

[B11] Etile E Despland E (2008) Developmental variation in the forest tent caterpillar: life history consequences of a threshold size for pupation. Oikos 117: 135-143. doi: 10.1111/j.2007.0030-1299.16114.x

[B12] Hopkin SP Read HJ (1992) The Biology of Millipedes. Oxford University Press, Oxford, 233 pp.

[B13] Jeekel CAW (1968) On the Classification and Geographical Distribution of the Family Paradoxosomatidae (Diplopoda, Polydesmida). Bronder-Offset, Rotterdam, 165 pp.

[B14] Kime RD Enghoff H (2011) Atlas of European Millipedes (Class Diplopoda). Vol. 1. Orders Polyxenida, Glomerida, Platydesmida, Siphonocryptida, Polyzoniida, Callipodida, Polydesmida. Pensoft, Sofia, 282 pp.

[B15] Kingsolver JG (2007) Variation in growth and instar number in field and laboratory *Manduca sexta*. Proceedings of the Royal Society B 274: 977-981. doi: 10.1098/rspb.2006.0036 17251106 PMC2141666

[B16] Mesibov R (2011) External anatomy of Polydesmida. http://www.polydesmida.info/

[B17] Sahli F (1968) Le développement post-embryonnaire des Polydesmides et la question de l’existence d’une périodomorphose chez ces Diplopodes. Bulletin Scientifique de Bourgogne 25: 309-332.

[B18] Shelley RM (2000) Revision of the milliped subfamily Desmoninae (Polydesmida: Sphaeriodesmidae). Myriapodologica 6: 27-54.

[B19] Stephenson JW (1961) The biology of *Brachydesmus superus* (Latz.) Diplopoda. Annals and Magazine of Natural History (Ser. 13) 3: 311–319.

[B20] Verhoeff KW (1916) Abhängigkeit der Diplopoden und besonders der Juliden-Schaltmännchen von äußeren Einflüssen. Zeitschrift für Wissenschaftliche Zoologie 116: 535-586.

[B21] Verhoeff KW (1923) Periodomorphose. Zoologischer Anzeiger 56: 233-254.

[B22] Verhoeff KW (1928) Durch Zucht erhaltene Formen des *Polydesmus complanatus, illyricus* Verh. und ihre Bedeutung, sowie Beurteilung der Elongation. Zeitschrift für Morphologie und Ökologie der Tiere 12: 684-705. doi: 10.1007/BF00403123

